# Data-driven epidemiologic approach to conducting site feasibility for a global phase III tuberculosis vaccine clinical trial

**DOI:** 10.1371/journal.pgph.0002544

**Published:** 2023-11-08

**Authors:** Wai-Ling Mui, Falgunee K. Parekh, Ashley S. Tseng, Joy Toro, Taylor Craig, Maggwa Ndugga, Alexander C. Schmidt, Alemnew F. Dagnew, Craig Penz, Ghiorghis Belai

**Affiliations:** 1 EpiPointe, Cary, North Carolina, United States of America; 2 FHI Clinical, Durham, North Carolina, United States of America; 3 Bill & Melinda Gates Medical Research Institute, Cambridge, Massachusetts, United States of America; Centro de Investigación y Asistencia en Tecnología y Diseño del Estado de Jalisco: Centro de Investigacion y Asistencia en Tecnologia y Diseno del Estado de Jalisco, MEXICO

## Abstract

An efficacious tuberculosis (TB) vaccine is critical to reducing the global burden of TB. TB vaccine trials require the identification of multiple sites globally that have both a high incidence of TB and the capacity to conduct a clinical trial. To expand the diversity of potential phase III TB vaccine trial sites to be considered for inclusion, we describe a novel epidemiologic method that incorporates approaches from a variety of public health practices. Our approach incorporates analytic methodology to enable quantification and validation of qualitative information from disparate data sources, and epidemiologic analysis to systematically assess site-specific TB epidemiology. The integration of robust data-driven practices, and more quantitatively focused analysis, allowed for the objective evaluation of sites, which resulted in the identification of sites and catchment areas with high TB burden that may not have been previously considered. This suggests that an integrated epidemiologic methodology, not traditionally utilized for clinical trial site evaluations, could be integrated into site feasibility assessments as it results in more rapid site identification and reduces unintended bias.

## Introduction

Tuberculosis (TB) remains one of the greatest global health challenges with approximately 10 million cases reported to the World Health Organization (WHO) each year [1]. There are a number of TB vaccine strategies currently in development including a protein subunit adjuvanted vaccine, revaccination with BCG, inactivated whole cell non-tuberculosis mycobacterium, live, genetically attenuated, and most recently a mRNA-based vaccine [2–4]. While mass vaccination with the current TB vaccine, Bacillus Calmette-Guerin (BCG), has been effective in protecting against the most severe forms of childhood TB, the efficacy wanes in adolescence and provides virtually no protection against adult-type pulmonary TB [5]. A recent study conducted in previously uninfected South African adolescents demonstrated a BCG revaccination efficacy of 45% against prevention of sustained infection [6]. Another recent study has shown an efficacy of 49.7% (95% confidence interval (CI), 2.1% to 74.2%) for the M72/AS01E vaccine candidate against developing bacteriologically confirmed pulmonary TB disease in adults with Mtb infection (IGRA positive) at vaccination, and no safety signals that would prevent further development of the vaccine candidate were observed [7]. An efficacious TB vaccine is critical to combatting the TB epidemic and reducing global burden to achieve the WHO End TB strategy targets of reducing TB deaths by 95% and new cases by 90% by 2035 [8].

While several successful phase II studies of TB vaccines have paved the way for development of global registrational phase III clinical trials [2], the development of such a trial requires careful planning, starting from site feasibility with identification of sites with high incidence of TB, in order to ensure that vaccine efficacy endpoints can be met with a reasonable sample size and within a reasonable duration of follow up. Furthermore, there are a number of factors that may impact vaccine efficacy, including HIV/TB co-infections and Tuberculosis Preventive Treatment (TPT) implementation that must be taken into consideration in the design, size, and analysis of a phase III trial [2]. In addition, we must consider operational aspects such as storge and administration of investigational product. Taking the different factors into account, successful evaluation of TB vaccine efficacy in a phase III trial will require a sample size that is much larger than a phase IIb study and may require enrollment of 10,000 or more participants per study arm [2].

Therefore, a phase III trial requires identification of a large number of clinical trial sites that recruit from communities with very high TB incidence to ensure sufficient enrollment of participants and adequate capacity to sustain the long follow-up time vaccine efficacy studies require. Identification of a sufficient number of quality sites is pivotal to ensure efficacy endpoints are met, however, traditional mechanisms of assessing site feasibility require a resource-intensive approach. Typically, for industry-sponsored clinical trials, timelines are tight and contract research organizations (CROs) have limited time and budget to determine the optimal strategies to target the research population. Most CROs implement a site feasibility assessment process using a questionnaire template that may or may not be customized for the specific study. To rapidly identify capable sites, there is also a tendency to revisit the same clinical research sites and seek information from familiar investigators or Key Opinion Leaders (KOLs), which limits the potential reach of studies. Additionally, the important epidemiologic risk factors of disease transmission for those sites are not evaluated in a systematic way that can inform clinical trial design and impact the incidence of efficacy endpoints [9]. As a result, studies may not target populations with high burden of disease due to limited resources, time, and visibility.

We combined methods across epidemiology, public health analysis, and data science to develop an expanded approach to site feasibility that incorporates an integrated assessment of disease epidemiology and more in-depth source evaluations and data validation which can impact the type of information that informs overall site feasibility. A combination of these methods can be applied to gather a sustained understanding or situational awareness of a specific disease, or biological event, In tandem, these analytic practices can also be utilized to identify and assess the individual factors within a given region, community, or population that impacts the degree and severity of disease transmission relevant to a location [10]. Moreover, the ability to turn qualitative, normally highly-subjective data (i.e., site feasibility assessment information), into quantifiable disease-related indicators results in a more focused and granular understanding of a region’s burden of disease [11]. We integrated a systematic epidemiologic methodology with traditional site feasibility processes to rapidly identify catchment areas considered hotspots of TB transmission and streamline the evaluation of essential site capacity data for a global phase III TB vaccine trial. Here, we describe our methodologic approach.

## Materials and methods

**Fig 1:**
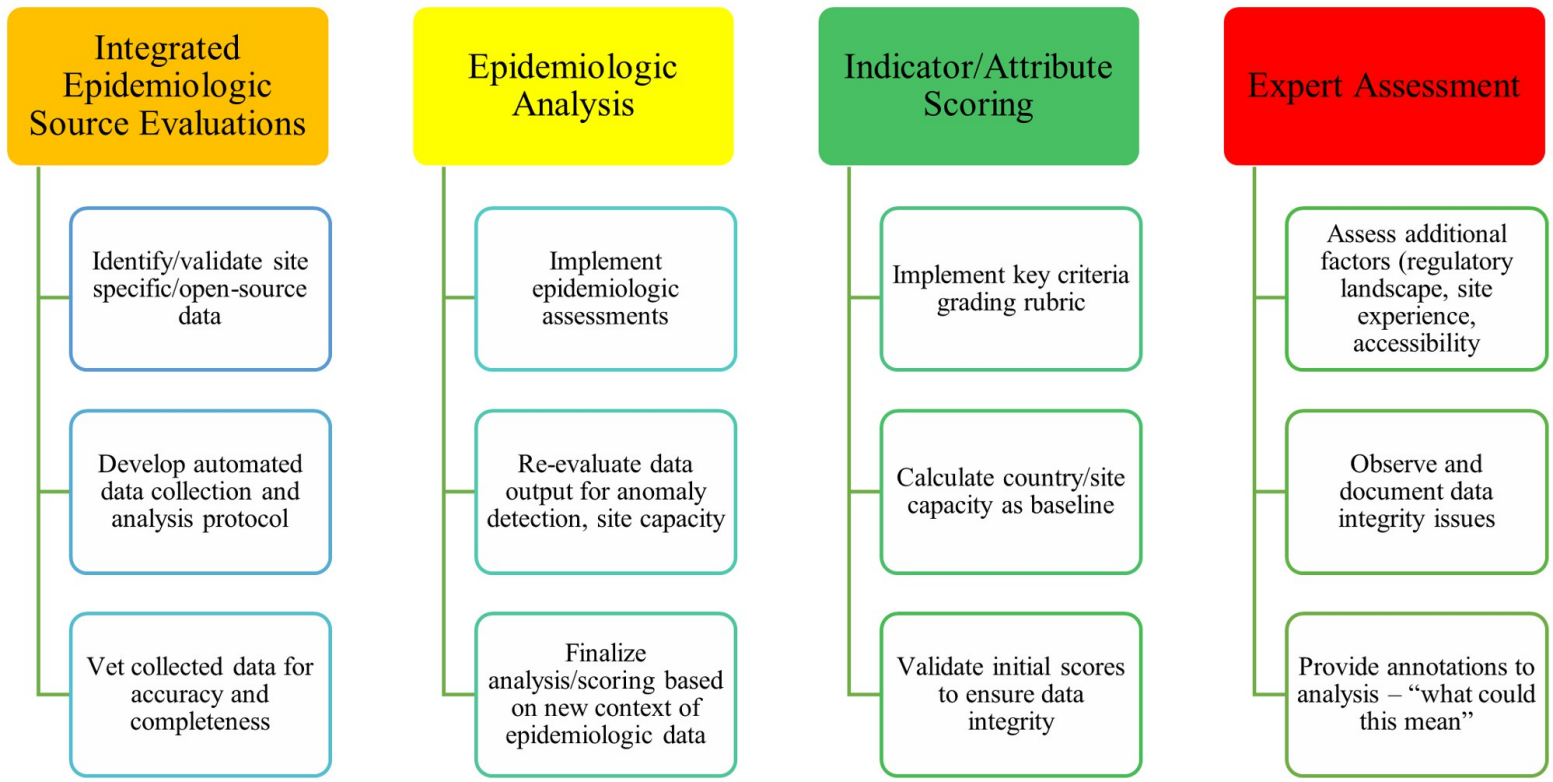
Major components of integrated epidemiologic assessments. Detailed description of the four components of the methodology: 1) integrated epidemiologic source evaluations, 2) epidemiologic analysis, 3) indicator/attribute scoring, and 4) expert assessment.

A study site selection for an epidemiologic study in preparation for a phase III clinical trial effort with a target population of healthy 15–34-year-olds at high risk of TB infection served as the foundation for our research. To systematically evaluate a large expanse of possible Phase III sites, we integrated epidemiologic assessments with traditional site feasibility processes to rapidly aggregate, vet, and analyze site-specific data. We also incorporated precise epidemiologic assessments to improve situational awareness of TB-related disease burden. Overall, the methodology comprised of four major components: 1) integrated epidemiologic source evaluations, 2) epidemiologic analysis, 3) indicator/attribute scoring, and 4) expert assessment (Fig 1). To facilitate the implementation, we developed a customized Feasibility Assessment Tool (FAT) to enable consistent collection of disparate individual site feasibility data.

### Site identification (Key Criteria)

Based on pre-existing knowledge, expertise with TB clinical trials, and WHO TB data [1], we pre-identified 221 sites in 25 WHO high-burden countries to apply our integrated methodology (Fig 1). Five Key Criteria were defined to identify known challenges and disease-related factors that most critically impact a site’s ability to successfully meet vaccine efficacy endpoints. Key Criteria included: *Incidence*, *Site Capacity*, *Regulatory Landscape*, *Experience*, *and Accessibility* (Table 1). Each was weighted *a priori* by clinical trial subject matter experts according to their relative impact on a successful clinical trial outcome. In our assessment, Incidence was the most heavily weighted criterion due to the importance of meeting vaccine trial endpoints. For a phase III TB vaccine trial, evaluating national-level incidence would not be sufficient; therefore, it was imperative to identify local catchment areas that were hotspots of TB transmission to enable enrollment of a sufficient number of high-risk individuals for evaluation of vaccine efficacy. Site Capacity was the second most heavily weighted criterion to ensure that a site had the proper resources in place to manage a phase III TB trial or had the potential to build capacity in order to sustain the needs of a clinical trial. Since Incidence and Site Capacity were the most heavily weighted criteria, this paper focuses on the methods used to assess these two criteria in greater detail.

**Table 1:** Key Criteria in site feasibility.

Key Criteria	Weight	Criteria Definition
Incidence	30	Known local and catchment area TB burden/prevalence within the target population above 300/100,000 population
Site Capacity	25	Capability to manage a phase III TB trial, lab access to collect and ship variety of samples, proper equipment, infrastructure to support study
Regulatory Landscape	15	Country-level clinical trial application filing requirements, submission approval timelines, regulatory requirements
Experience	15	Ability to recruit participants, oversee activities to maintain study, previous familiarity with population-based vaccine trials
Accessibility	15	Geographic accessibility, logistics/supply chain challenges, political instability
**TOTAL**	100	

**Fig 2:**
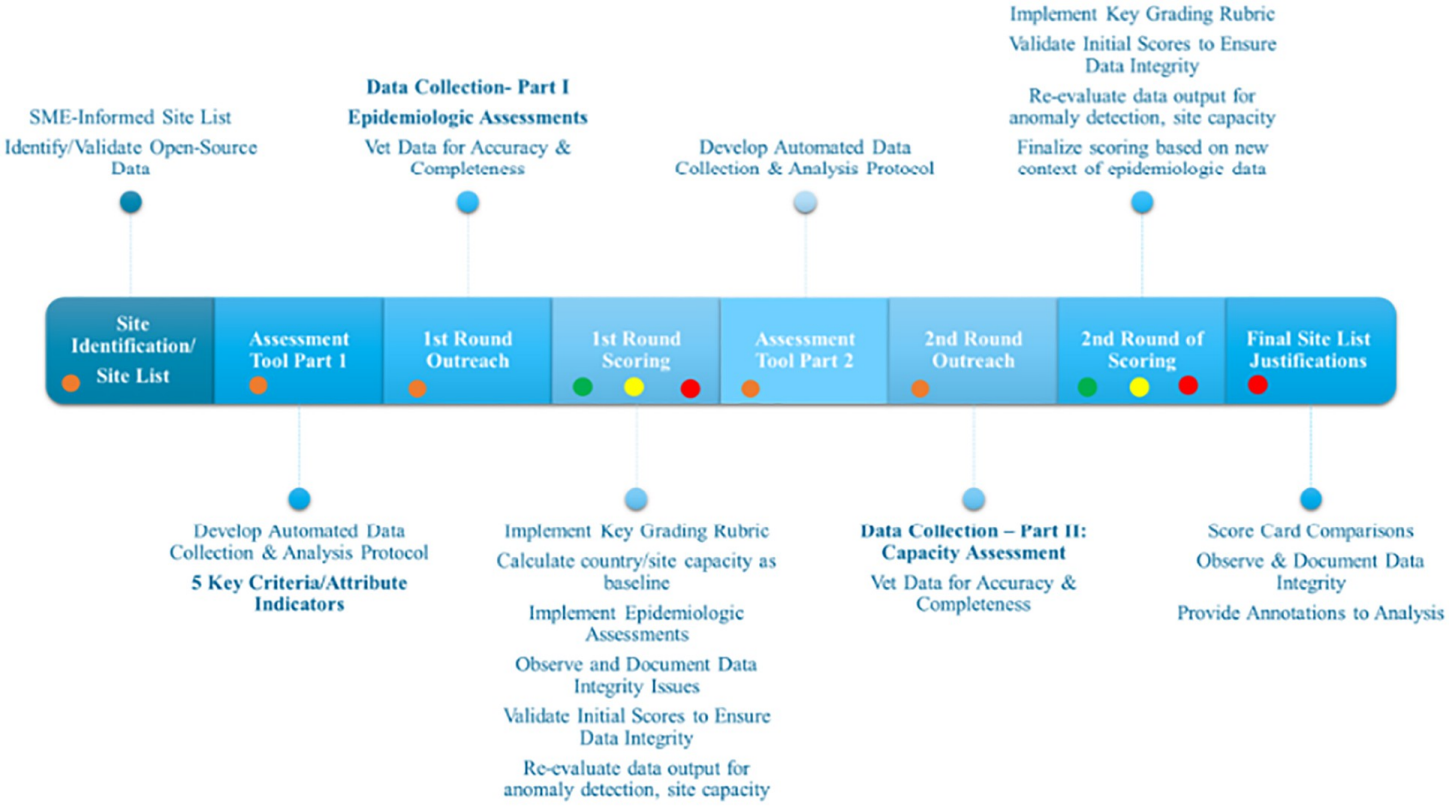
Integrated epidemiologic methodology and site feasibility process. Description of process followed to integrate our epidemiologic methodology to the traditional site feasibility assessment to enable a more systematic and objective approach to evaluating potential sites for the phase III clinical trial.

Based on the weighted criteria we combined the four major components of our integrated epidemiologic methodology into the traditional site feasibility process. Fig 2 describes the process followed, which includes a systematic and consistent approach to data collection, data validation, epidemiologic analysis, and expert assessment.

### Integrated analytic indicators and attributes

Incidence and Site Capacity key criteria were further refined according to specific indicators (or factors) that influenced a site’s eligibility for inclusion in a clinical trial. Borrowed from traditional biosurveillance practice, which is loosely defined in epidemiologic practice as the act of surveying factors of a biological event to inform public health practice, ‘Indicators’ are the pre-defined factors that serve as the basis for extraction of relevant event-based data [11]. As such, we identified key indicators relevant to evaluating a site’s ability to conduct a trial. These indicators were Incidence Rate(IR), Capacity(CP), Timeliness(TL), and Completeness(CO). Indicators were assigned quantitative ‘scores,’ or numerical grades, to normalize collected data and ensure uniform analysis and scoring per traditional biosurveillance methodological practices [12] (i.e., categorization of events and their elements into specific indicators that provide context to an event of interest). IR and CP indicators were further delineated into attributes.

#### Incidence rate

This indicator, meant to access a site’s level of TB burden, was further divided into smaller attributes for evaluation. These included Reported Rate (IR1) of TB among population size 15–34-year-olds for years 2017, 2018, 2019, and 2020; Accuracy (IR2) of data as determined by study target population data, number of years of data provided, and if any additional TB prevalence data was provided (e.g., results of a prevalence study); Granularity (IR3) regarding specificity and characterization of the study target area; and Justification (IR4) of TB data based on the type of evidence a site was able to provide to validate TB frequency and incidence (e.g., local notification reports, treatment registers, laboratory registers, or clinic facility reports).

#### Capacity

The Capacity indicator was also further divided into attributes for evaluation, including Study Area Capacity (CP1), TPT Policies (CP2), and Lab Capacity (CP3) of the site. Study Area Capacity was based on reported proximity to international travel and/or shipping facilities; sites that were located in urban or peri-urban areas were more likely to have international travel and shipping capabilities nearby, and thus were awarded a higher score. TPT Capacity assessed reported current and planned updates to national TPT guidelines as site-specific TPT policies and implementation can impact the design and conduct of the clinical trial and evaluation of vaccine efficacy. Lab Capacity assessed necessary experience in collecting and processing blood and sputum samples, understanding of international lab shipping protocols, and ability to utilize nearby labs to expand capacity.

#### Timeliness

Timeliness assessed the response times of potential study sites. Sites were scored higher if the turnaround time to provide their site information was 5 business days or less. Sites that took more than 10 business days to respond were differentiated from sites that did not respond at all to acknowledge the effort of these sites. Sites that were able to successfully provide the requested information, even if it was beyond the anticipated time frame, were awarded Timeliness points for taking the time to respond.

#### Completeness

Completeness evaluated the robustness of data collected from sites, including their ability to provide necessary supplemental information reflecting their epidemiological, laboratory, and implementation capacities.

### Grading rubric

The Incidence-related questions were given a total weight of 60, the Site Capacity-related questions were given a weight of 20, and Timeliness and Completeness each were assigned a weight of 5, resulting in possible total score of 100. Questions were assigned to each attribute, sub-weighted, and assigned scoring options associated with those weights (Table 2).

**Table 2:** Indicator, attributes, questions and scoring.

Attribute	Questions	Scoring Options	Score
**Incidence Rate**			
IR1: Reported Rate	Average reported TB case notification data for 2017–2020	Range 1: >700	15
		Range 2: 500–699	13
		Range 3: 250–499	11
		Range 4: <250	0
	Specific TB hotspot(s) reported within study target area	Yes	5
		No	0
IR2: Accuracy	Estimated population of study target area provided	Yes	5
		No	0
	Number of years of data provided	Point for each year (max 4)	1–4
	Provided additional prevalence data	Yes	5
		No	0
IR3: Granularity	Description of study target area	Informative	Informative
	Characterization of study target area	Urban	5
		Peri-Urban	4
		Suburban	3
		Rural	2
		Other	1
	Characterization of TB hotspots	Urban	5
		Peri-Urban	4
		Suburban	3
		Rural	2
		Other	1
IR4: Justification	Types of evidence of TB frequency provided	TB Notification Report	1
		TB Treatment Register	1
		TB Laboratory Register	1
		Clinic Facility Report	1
		Other	*Based on SME*
	Provided evidence of TB frequency (URL or attachment)	Yes	5
		No	0
	Types of evidence of TB incidence provided	Scientific Publication	1
		Survey	1
		Other	*Based on SME*
	Provided evidence of TB incidence (URL or attachment)	Yes	5
		No	0
**Capacity**			
CP1: Study Area Capacity	Characterization of study target area	Urban	5
		Peri-Urban	4
		Suburban	3
		Rural	2
		Other	*Based on SME*
CP2: TPT Policy	National TPT Guidelines and Updates	Completed	5
		Not Completed	0
CP3: Lab Capacity	Experience with blood samples	Yes	4
	Experience with sputum samples	Yes	4
	Experience with international shipping of lab samples	Yes	4
	Experience with QuantiFERON-TB processing	Yes	4
	Experience working with a nearby lab for sample testing	Yes	4
**Timeliness**			
TL1: Timeliness	Number of business days to questionnaire completion	< = 5 days to complete	5
		6–10 days to complete	3
		>10 days to complete	2
**Completeness**			
CO1: Complete	Number of answers completed	All answered	5
		< = 3 questions left blank	3
		>3 questions left blank	1
**TOTAL SCORE**			**100**

TB, tuberculosis; TPT, Tuberculosis Preventive Treatment.

### Feasibility Assessment Tool (FAT)

We designed an initial FAT to collect, vet, and evaluate site-specific data related to Incidence and certain attributes of Site Capacity. The FAT was developed using Smartsheet’s ‘Forms’ capability, a collaboration and work management software tool that can be used to collect and assess large amounts of contextual data via a digital questionnaire [13]. We are sharing the collected response data as an MS Excel spreadsheet, which is more widely accessible globally, as supporting information (S3 Appendix). As Incidence was the most heavily weighted and critical criterion for initial consideration of a site, all of the Incidence attributes were evaluated in this initial FAT. However, only key attributes of Site Capacity necessary for further consideration of the site were evaluated in the initial FAT. Specific questions were developed in alignment with the Incidence and Site Capacity criteria, indicators, and attributes, and their corresponding weights. A questionnaire was developed and sent to the sites to collect data based on the key criteria. We aggregated summary data from potential clinical trial sites to inform our analysis. These data included, total population that the site served, site-specific population information for 18–34-year-olds, total number of TB cases reported per sites (i.e., TB log book case counts). No private or individual patient data, information or medical records were collected or used for this analysis.

#### Data collection (initial outreach)

As data were received via a completed questionnaire, it was automatically input into the Smartsheet database for evaluation. Additional metadata collected included tracking dates sent, received, geographic locations, and points of contact for each site.

#### Data validation

We developed a standardized workflow (S1 Appendix) for assessing each site to ensure consistency in validating data, clarifying ambiguity of data, and identifying gaps or errors that were specific to a location or demographic. Data definitions were normalized across regions to ensure uniform comparison of disparate sites. Necessary follow-ups, often including requests for additional information, allowed for more accurate scoring while strict workflows guaranteed equitable objective assessment across disparate sites.

#### Epidemiologic analysis

We conducted an assessment of Incidence based on the data provided by each site (as described in S2 Appendix). We also evaluated any additional forms of data provided (e.g., case notification, TB log books, prevalence survey, published articles) to assess consistency of data and to obtain a comprehensive understanding of the epidemiologic context of an individual site. Finally, the epidemiologic analysis allowed for rapid flagging of possibly erroneous or unexpected/anomalous data for follow-up.

#### Indicator and attribute scoring

We established a grading rubric which applied points to each question associated with the pre-established weighting of the attributes and indicators (Table 2). As data were collected, analysts conducted initial scoring based on this grading rubric within 24 hours. Epidemiology and public health experts then conducted quality assurance review of the initial score, identified and documented irregularities or issues, and sent requests for additional information or clarifications to the site. When sites provided additional information/clarifications, we followed the same process of initial scoring and quality assurance review.

#### Expert assessment

All epidemiologic and capacity data collected were assessed by a multi-disciplinary team comprised of epidemiology, clinical trial, and public health experts prior to finalizing the site score. The expert assessment included observation and documentation of any data integrity issues. In addition, the team provided annotations to the analysis to assist in interpretation of the data (e.g., “what could this mean?”).

### Inclusivity in global research

Additional information regarding the ethical, cultural, and scientific considerations specific to inclusivity in global research is included in the Supporting Information (S1 Questionnaire).

## Results

Promising sites were identified, and then further evaluated in a subsequent phase of the study (not described here) where Site Capacity, Regulatory Landscape, Experience, and Accessibility specific criteria would be evaluated.

The initial FAT was sent to 221 sites in 25 countries across three continents. Of these, 191 completed the FAT for scoring. Some countries, such as South Africa and the Philippines were considered priority countries due to their high TB burden and previous experience in conducting TB vaccine trials. While we still assessed and scored sites in these countries (South Africa [n = 27 sites] and the Philippines [n = 8]), using our systematic method, these sites were ultimately selected for further evaluation based on their previous experience with conducting TB vaccine trials in high incidence areas, which was considered priority for the clinical trial. More than half of sites (51%) that completed the FAT required at least one follow-up that included clarification questions or requests for additional information that would allow for more accurate scoring. Using our methodology and workflow for scoring, it took us approximately 10 weeks to collect responses, score, conduct expert assessment, follow-up with sites, and finalize scores for all 191 sites.

Site scores ranged from 40–92 out of a total 100 points (n = 191) (Table 3). Sites that scored >75 points were able to provide specific incidence or case notification data for a defined study target area for multiple years, met the necessary minimum incidence (>300 TB cases per 100,000 population), was able to provide some justification to support the epidemiology data reported, was located in an urban or peri-urban area, and had the necessary laboratory capacity required to process clinical trial samples and ship to out of country central laboratory. Those that scored >85 points were able to provide incidence or case notification data for a defined study target area for 4 years, provided multiple types of justification, were located in an urban area, had the necessary laboratory capacity, answered all the questions, and completed the FAT quickly (in ≤5 days). A number of sites that scored between 65–75 were often able to provide specific incidence or case notification data, had the necessary incidence, and laboratory capacity. However, the lower scores were often a result of gaps in the information provided; for example, limited amount of justification, not providing TPT guideline information, or delays in completing the FAT. Delays were often related to issues with obtaining the data due to the COVID-19 pandemic. In general, sites that scored >65 were identified for further evaluation based on their Site Capacity, Regulatory Landscape, Experience, and Accessibility information, in a second phase not described in this paper. Using our methodology, we were able to rapidly identify a total of 92 sites from 23 countries that met the epidemiologic and basic capacity requirements, excluding sites in South Africa and the Philippines- which were already selected for further evaluation based on their previous experience conducting TB vaccine trials in high incidence areas.

**Table 3:** Score values and descriptions.

Site Scores^a^	Scoring Description	Number of Sites in Score Range
**≥85**	Provided incidence for specific catchment area for 4 year and multiple types of justification; had high TB incidence; located in urban area (near airport), had necessary lab capacity, answered all questions; completed FAT in ≤ 5 days	14
**75–84**	Provide incidence for specific catchment area for multiple years, met minimum incidence (>300/100,000); provided some justification; located in urban or peri-urban area; had necessary lab capacity	54
**65–74**	Provide incidence for specific catchment area for some years; had minimum incidence; provided limited justification; had necessary laboratory capacity; not providing TPT information	45
**<65**	Did not meet minimum incidence; provided limited or no justification	80
**Total**		193 ^b^

^a^ Site scores ranged from 40–92 out of total 100 points for the 191 sites evaluated.

^b^ The discrepancy in the total number of tallied sites per country is due to the fact that some sites (e.g., Uganda), were split into 2 catchment areas, with the same site investigator (e.g., UGA-002a, UGA-002b) so that if counted individually, would total 193.

In addition to identification of promising sites based on a systematic and quantitative evaluation, our methodology also allowed us to obtain a more granular understanding of the epidemiology of TB that was highly informative in identifying specific catchment areas. TB incidence reported at the national level may not necessarily reflect the local epidemiologic situation. We found that often local case notification or incidence calculated from site-specific data was higher than national TB incidence reported by WHO. Table 4 shows the local TB incidence calculated using our methodology based on data provided by local sites and the national-level TB incidence reported by the WHO for each country for 2017–2020. Brazil, the Democratic Republic of the Congo (DRC), India, and Peru are all examples of locations where the TB incidence calculated using our methodology based on data provided by local research sites was higher, sometimes significantly, than the national-level TB incidence data reported by the WHO. In Pakistan, the average incidence calculated by our team was close to the WHO national incidence; however, the difference between the minimum and maximum incidence rates calculated by our team were much larger.

**Table 4:** Tuberculosis calculation examples: World Health Organization (WHO) reported national incidence [14] versus local incidence.

Country	Source	2017 Incidence (Min.-Max.)	# of Sites (Local)	2018 Incidence	# of Sites (Local)	2019 Incidence	# of Sites (Local)	2020 Incidence	# of Sites (Local)
**Brazil**	WHO	44 (38–51)		46 (39–53)		46 (39–53)		45 (38–52)	
	Local	187 (135–427)	4	253 (145–1,311)	5	274 (144–1,404)	6	191 (130–337)	4
**DRC**	WHO	322 (208–461)		321 (207–459)		320 (207–457)		319 (206–456)	
	Local	417 (280–1303)	10	1,013 (312–16,536)	22	1,006 (109–12,828)	22	1,203 (83–8,228)	24
**India**	WHO	204 (136–286)		199 (134–276)		193 (132–266)		188 (129–257)	
	Local	1,085 (38–1,091)	3	627 (38–1,487)	8	1,000 (40–1,899)	9	442 (26–2,003)	5
**Kenya**	WHO	319 (175–505)		292 (170–446)		267 (163–396)		-	
	Local	90 (70–111)	2	110 (109–112)	2	84 (66–308)	3	67 (52–308)	3
**Pakistan**	WHO	267 (188–359)		265 (187–356)		263 (187–353)		259 (185–346)	
	Local	165 (10–896)	21	155 (9–1,240)	21	165 (9–1,080)	21	132 (10–1,480)	21
**Peru** ^a^	WHO	119 (91–150)		119 (91–150)		119 (91–150)		116 (87–149)	
	Local	296 (167–412)	6	327 (130–389)	4	342 (136–1,616)	6	119	1

^a^Site incidence multiplied by missing notification factor (gap factor) [1] to estimate actual incidence—1.19–1.21.

## Discussion

The burden of TB has only been exacerbated by the COVID-19 pandemic due to disrupted case finding and treatment strategies, and the case numbers of TB are expected to increase substantially over the next several years [15, 16]. The systematic incorporation of an integrated data-driven epidemiologic methodology into a traditional TB site feasibility assessment laid the groundwork for maximizing the success of a future phase III TB vaccine clinical trial. It allowed us to expand the breadth and depth of site feasibility assessments. Through our integrated methodologic process, we were able to rapidly gather extensive and detailed information on TB incidence within specific catchment areas for 191 sites in 25 countries in 10 weeks. This included the development of the FAT and database to collect and analyze the data.

The level of detail and the granularity of the information we collected went well beyond a typical feasibility study. Our method enabled objective assessment of layers of data that included not only incidence data, but also epidemiological factors, demographic, and location/capacity-related information. This allowed us to conduct a deeper evaluation of TB burden at the local level not traditionally considered in site feasibility evaluations. We compared site value across disparate regions and locations in an objective, quantitative manner (e.g., via scores and points) that improved justification and decreased overall bias in site selection for a trial. In addition, the Score Cards could be sorted by various factors, such as by country, indicator, or attribute.

The totality of a country’s site data serves as a justifiable means for referencing country-level factors that may influence clinical trial development. We found that site score comparisons at the country level were highly relevant to site selection because we could assume that similar extenuating factors contributed to incidence and reporting of TB case notification data. This helped rapidly determine which sites within a country were most promising for inclusion and therefore merited further assessment. For example, DRC was not originally considered a high priority country to include in the trial; however, there were several sites in the country with very high TB incidence. This, combined with high scores in the feasibility assessment, demonstrated that sites in DRC have both the incidence and capacity and should be considered for inclusion in the trial. In contrast, Kenya has historically been known to have high TB burden; however, the data provided showed that the sites in Kenya had lower TB incidence than expected, likely because of improvement in early detection and treatment of active TB cases.

The incidence of TB is heterogeneous within a country and a beneficial outcome of this methodology was that it enhanced the equitable assessment of sites by focusing on site level data. CROs usually conduct site feasibility by reaching out to known sites, investigators, or KOLs which is not an optimal approach in identifying sites for Phase III TB vaccine clinical trials. Clinical trials often are not available or accessible to sites that work with underrepresented populations. These populations are often at the greatest risk of infectious diseases like TB and could benefit greatly from having access to novel therapeutics and interventions. Clinical trials are integral to improving and sustaining equity related to high burden of disease, yet <20% of trials are conducted in LMICs or developing countries [12]. In addition to the individual and public health benefits, sites that serve these underrepresented populations may also have an opportunity to enhance their capacity and capabilities through the conduct of a clinical trial. It is well-established that improving equity in clinical trials is necessary both in terms of ensuring that the most vulnerable populations who often experience the greatest burden of disease have access to clinical trials and that a diversity of populations are represented in clinical trials [17–19]. In addition, the lack of accessibility and under-representation of LMICs in global clinical trials contributes to the continued overall health inequities in these countries [12]. While there is increasing discussion on the importance of improving equity and accessibility of clinical trials to under-represented populations, the practical implementation of this remains a challenge.

Notably, utilization of this method for site feasibility required a multi-disciplinary team of epidemiological and clinical trial experts with relevant experience in the fields of infectious disease transmission and public health. Input from these experts, in conjunction with data analysts and public health experts, ensured the information was assessed within the appropriate epidemiologic context.

Thus, we conducted this site feasibility study in an objective manner that reduces resource demands, enhanced the ability to objectively evaluate vital epidemiological data, and expands diversity of sites evaluated. Our novel approach accounts for variability across target populations, geographies, and capacities, ultimately striving for comparability in epidemiological measures reported across potential sites. Moreover, the use of standardized evaluation protocols increased confidence in the final grading and scoring of sites that was conducted in a time and cost-efficient manner.

Our application of an integrated epidemiologic methodology to site feasibility allows for the operationalization of equity and accessibility to clinical trials. The rigorous assessment of indicators and attributes of a site’s epidemiology and capacity resulted in a systematic and objective evaluation of sites that reduced the influence of bias. We found that some sites had significant incidence and basic site capacity that could potentially yield robust results for a clinical trial but would have been unlikely to be considered through traditional site feasibility assessment methods. Using a systematic approach like the integrated epidemiologic methodology described here expanded the range of sites serving underrepresented populations and help provide more equitable access to life-saving therapeutics.

## Supporting information

S1 Questionnaire Inclusivity in global research questionnaire. (DOCX)

S1 Appendix TB site feasibility questionnaire: Scoring workflow. (DOCX)

S2 Appendix Calculating Incidence Rate (IR). (DOCX)

S3 Appendix Source data. (XLSX)
