# Supplementary material for: Data-driven epidemiologic approach to conducting site feasibility for a global phase III tuberculosis vaccine clinical trial
Source: PLOS Glob Public Health. 2023 Nov 8;3(11):e0002544. doi: 10.1371/journal.pgph.0002544 (PMC10631637; doi:10.1371/journal.pgph.0002544)
Supplement: S1 Appendix — (DOCX) [file pgph.0002544.s002.docx]

**S1 Appendix: TB Site Feasibility Questionnaire: Scoring Workflow**

**Data Collection.** The TB epidemiology study site feasibility questionnaire was administered using *Smartsheet*- a collaboration and work management software tool. At the study onset, each potential site was sent an outreach email with a summary of the study objectives and a direct link to the digital questionnaire. After a potential site completed the questionnaire, the Smartsheet auto-collated responses into a single spreadsheet. Within the spreadsheet, each question was represented as a column and each individual response was represented as its own row (supplemental appendix 3). This direct Smartsheet survey-to-spreadsheet capability reduced the likelihood of transcription errors and allowed for the consistent collection and assessment of site data.

**Initial Response Review.** Once questionnaire emails were sent, one team member was charged with monitoring the project Smartsheet on a daily basis. The response review team was comprised of two epidemiologists and one biosurveillance subject matter expert. As responses were collected, the designated team member would log initial response metadata (e.g., date/time collected, site location, etc.) and conduct a cursory review of the responses to ensure compliance (e.g., no duplicates or technical errors). Once an incoming response was verified, the team member would assign a “Site ID” number to it based on the ISO 3166-1 alpha-3 codes that are used to represent countries and dependent territories. Corresponding numbers were assigned in ascending order based on when the response was submitted. For example, “BRA-001” indicates the first questionnaire response submitted for a site in Brazil, and “BRA-002” indicates a second response submitted for a second site in Brazil. Site IDs were carried over to other project contexts to ensure consistency when referencing potential sites.

**Scoring Status.** Once a Site ID was assigned, a team member was appointed to begin round 1 of the scoring process, and a “score status” was marked. Red indicated the start of the scoring process (e.g., round 1). For quality assurance purposes, each questionnaire response was scored twice, where round 1 and round 2 were scored by different team members. The score statuses were marked yellow if round 1 of the scoring process had been completed, or if the team was following-up with a site for further information (i.e., further clarification was needed regarding a response). A green score status indicated that round 2 of the scoring process had been completed, and any necessary follow-up had been completed (i.e., a final questionnaire score could be reached).

**Scorecards.** To quantitatively assess each site’s response, points were given based on site responses in the Smartsheet where responses were assigned a certain point value based on the scoring rubric criteria (Table 2). A “scorecard” template (Table 2) was used to standardize how team members allocated points using the rubric. Scorecards were maintained on a Microsoft Excel spreadsheet where each tab of the spreadsheet corresponded to a country’s ISO 3166-1 alpha-3 code and each site’s individual scorecard was organized adjacently (left to right) in numeric order. Each site’s final score was the cumulative sum of points related to each response based on the pre-established weight of attribute and indicator criteria.

**Data Validation.** During the scoring process, to further ensure response data was accurately evaluated, scoring team members added follow-up notes into a specified column of the scorecard to document when further clarification was needed- or to document how ambiguous response data was validated. For example, if a site did not attach the necessary justification for data they submitted, a note was made to request the site provide proper documentation. When follow-up was needed, a site’s designated “project team contact”, was notified. They were responsible for relaying follow-up notes to sites and closing out requests. Internal notes were also logged on scorecards to highlight any anomalies identified during the scoring process. For example, if the scorer had to manually calculate population size (IR denominator) based on the provided target area population, or if a response did not accurately fit the scoring rubric and needed to be further discussed, scorers noted these issues in another separate column. Any such ‘adjudications’ were documented for reference in subsequent scoring determinations.

**Final Scores.** Final site scores from the questionnaire were used to determine eligibility to proceed to the second phase of site feasibility assessment (Table 3).
