# Supplementary material for: Data-driven epidemiologic approach to conducting site feasibility for a global phase III tuberculosis vaccine clinical trial
Source: PLOS Glob Public Health. 2023 Nov 8;3(11):e0002544. doi: 10.1371/journal.pgph.0002544 (PMC10631637; doi:10.1371/journal.pgph.0002544)
Supplement: S2 Appendix — (DOCX) [file pgph.0002544.s003.docx]

**S2 Appendix: Calculating Incidence Rate (IR)**

**IR Scoring Example**

Site DRC-010 reported TB case notification for all four years. For 3 years (2018-2020) the estimate was greater than 500 cases per 100,000, but the estimate was 280 cases per 100,000 population in 2017. According to the scoring rubric, an average TB incidence in the range of 250-499 cases per 100,000 population is awarded 11 points while an average TB incidence in the range of 500-699 cases per 100,000 population is awarded 13 points. Each year of TB incidence is weighted equally, but the team accounted for the 2017 outlier for DRC-010 and weighed it slightly greater, thus awarded the site 12 points for their IR score.

TB incidence was the most heavily weighted key criteria for the selection of a site for inclusion in the epidemiology study conducted in preparation for a Phase III vaccine trial. This is because recruitment of participants who are at high risk of TB exposure is necessary to meet vaccine trial efficacy endpoints, and therefore the identification of sites/catchment areas that have high TB incidence is critical. However, accurate assessment of TB incidence requires active case detection during long-term follow-up of very large populations and is therefore usually not feasible. Instead, most countries use and report case notifications of TB as a proxy for estimating TB incidence according to the World Health Organization (WHO)^^[[1]](#footnote-1)^^. We used case notification to estimate TB burden (here called Incidence Rate); some sites provided an estimate that incorporated the missing notification factor established by WHO for that country. This data was used to determine the final IR score and the missing notification factor that they multiplied by was noted.

We developed an approach to uniformly estimate TB burden across various reporting systems across study sites. It was important to develop a method that was aligned with the type of data that most countries already collect to facilitate rapid completion of the survey and enable best possible comparability across sites at a global level. TB case notification (inclusive of new and relapse cases) is a commonly used metric in reporting TB burden. We calculated the estimate among 15–34-year-olds based on a direct method of [(new & relapse cases/population) x 100,000]. Each site was asked for 1) the total number of new and relapse TB cases among 15–34-year-old individuals in their study target area and 2) the total population of 15–34-year-old individuals in their study target area. If a site did not provide the estimated 15-34 population for each year, then the team multiplied the UN estimated 15-34 population proportion^^[[2]](#footnote-2)^^ rounded to 3 decimal points by the study target area population size that was provided by the site in question 3 . The team divided the total number of new and relapse cases by the total population of 15–34-year-old individuals, then multiplied this proportion by 100,000 to approximate TB cases per 100,000 population. This process was repeated for up to four years of data provided by the site, 2017-2020.

We took an average (unweighted) of all years of the estimates to determine the incidence rate (IR) score. If there was an outlier in the estimates between years (i.e., significant increase/decrease in one particular year), then epidemiologic expert assessment and discussion among epidemiologists and TB subject matter experts (SMEs) were utilized to determine the site’s final IR score. All forms of data provided by the site (incidence, prevalence, TB register data) were evaluated using epidemiologic expert assessment to determine the final IR score. It should be noted that the incidences calculated are a best estimate of the TB burden for that setting. True incidence rates were not captured from the site feasibility questionnaires due to the nature of the project relying solely on locally available data on TB burden in catchment areas.

1. World Health Organization. (2020). Estimated epidemiological burden of TB in 2019 for 30 high TB burden countries, WHO regions and globally. [Table 4.3]. WHO Global Tuberculosis Report 2020. https://apps.who.int/iris/bitstream/handle/10665/336069/9789240013131-eng.pdf [↑](#footnote-ref-1)
2. United Nations. (2021). World Population Prospects Data Query. United Nations Department of Economic and Social Affairs Population Dynamics. https://population.un.org/wpp/DataQuery/. [↑](#footnote-ref-2)
